# Chemotherapy Options for Locally Advanced Gastric Cancer: A Review

**DOI:** 10.3390/cancers17050809

**Published:** 2025-02-26

**Authors:** Yuliya Semenova, Altay Kerimkulov, Talgat Uskenbayev, Dinara Zharlyganova, Oxana Shatkovskaya, Tomiris Sarina, Almira Manatova, Gulfairus Yessenbayeva, Tasbolat Adylkhanov

**Affiliations:** 1 Department of Surgery, School of Medicine, Nazarbayev University, Astana 010000, Kazakhstan; yuliya.semenova@nu.edu.kz; 2 Department of Multidisciplinary Surgery, National Research Oncology Center, Astana 020000, Kazakhstan; altay.kerimkulov@gmail.com (A.K.); uskenbaev_talgat@mail.ru (T.U.); tomiris.md@gmail.com (T.S.); adylkhanov.kz@mail.ru (T.A.); 3 Department of Scientific Management, National Research Oncology Center, Astana 020000, Kazakhstan; science.nroc@gmail.com (D.Z.); yessenbayeva.gulfairus@gmail.com (G.Y.); 4 Board for Strategic Development, Scientific and Educational Activities, National Research Oncology Center, Astana 020000, Kazakhstan; 1972arty@mail.ru

**Keywords:** gastric cancer, locally advanced, systemic chemotherapy, targeted therapy, intraperitoneal chemotherapy

## Abstract

Gastric cancer ranks as the sixth most common cancer globally and remains a significant contributor to cancer-related mortality. Detection at the stage of locally advanced gastric cancer (LAGC) offers an opportunity to improve survival outcomes through a multimodal treatment approach. Current management typically involves a combination of neoadjuvant and adjuvant systemic chemotherapy alongside gastrectomy. In select cases, the strategy is expanded to include targeted therapy and intraperitoneal chemotherapy (IPC), delivered via hyperthermic intraperitoneal chemotherapy (HIPEC) or pressurized intraperitoneal aerosol chemotherapy (PIPAC). While systemic chemotherapy and targeted therapies are implemented both preoperatively and postoperatively, IPC is often utilized postoperatively but can also be administered preoperatively or intraoperatively. Targeted therapy remains an area of active investigation in the LAGC setting, with promising agents targeting pathways such as HER2 and VEGF. However, the role of IPC modalities is less well established. The clinical utility of HIPEC remains controversial due to mixed outcomes in trials, and there is a paucity of clinical studies evaluating the preventive role of PIPAC. Future research is needed to elucidate the potential of novel targeted therapies and IPC modalities in the treatment of LAGC, particularly concerning optimal timing, frequency, and integration with systemic therapies. This review aims to summarize the current state of knowledge on chemotherapy options for LAGC, highlight existing gaps and controversies, and propose directions for future research.

## Simple Summary

Gastric cancer is the sixth most common cancer worldwide and a major cause of cancer-related deaths. For patients with locally advanced gastric cancer, combining treatments like chemotherapy and surgery can improve survival. Treatment often includes chemotherapy given before and after surgery, with some cases involving targeted drugs or special types of chemotherapy delivered directly into the abdominal cavity. This review explores current treatment options for locally advanced gastric cancer, emphasizing the need for more research to determine how best to use these therapies and integrate them into care. The ultimate goal is to improve outcomes for patients through better understanding and innovation in treatment approaches.

## 1. Introduction

Cancers represent a significant global health burden, affecting millions of individuals each year. According to the World Health Organization (WHO), cancer is the second leading cause of death worldwide, responsible for approximately 10 million deaths annually [1]. The global incidence of cancer is also rising, with an estimated 20 million new cancer cases reported in 2022. Gastric cancer is the fifth most common type of cancer worldwide following lung, breast, colorectal, and prostate cancers [2]. However, it is the third leading cause of cancer mortality, following lung and colorectal cancers [3]. Infestation with *Helicobacter pylori* is considered the major causative risk factor, and other known risk factors include advanced age and unhealthy diets [4].

Gastric cancer treatment typically involves a combination of surgery and chemotherapy, depending on the tumor stage, patient’s general health, and tumor-specific characteristics [5]. Resection is a surgical method applied for operable gastric cancers, leading to the physical removal of the tumor and adjacent lymph nodes, if indicated [6]. Neoadjuvant (preoperative) and adjuvant (postoperative) chemotherapy are used to reduce tumor size before surgery, eliminate microscopic residual disease after surgery, and improve overall survival rates [7]. These approaches can be combined with radiotherapy to enhance treatment outcomes [8].

Chemotherapy is a cornerstone of gastric cancer treatment, especially in advanced and locally advanced stages of the disease, and may be applied in various clinical settings. Neoadjuvant chemotherapy aims to shrink the tumor, making it more amenable to surgical resection and reducing the risk of micrometastases [9]. Adjuvant chemotherapy helps eliminate residual cancer cells after surgical resection and is often administered as a continuation of neoadjuvant chemotherapy, forming a perioperative chemotherapy approach [10]. Patients with inoperable or metastatic disease benefit from palliative chemotherapy, which alleviates cancer-related symptoms and extends survival [11]. Chemotherapy may also be combined with other treatment modalities, such as targeted therapy, to enhance therapeutic efficacy [12].

Aside from systemic chemotherapy, gastric cancer may be treated with intraperitoneal chemotherapy (IPC), which delivers chemotherapeutic agents directly into the peritoneal cavity, enabling higher local drug concentrations while minimizing systemic toxicity. IPC is primarily indicated for patients with peritoneal metastasis or those at high risk for peritoneal recurrence, particularly in cases where standard systemic therapy may not achieve sufficient local control [13]. Advances in intraperitoneal delivery techniques, including hyperthermic intraperitoneal chemotherapy (HIPEC) [14] and pressurized intraperitoneal aerosol chemotherapy (PIPAC), have demonstrated promising outcomes in improving drug penetration and therapeutic efficacy within the peritoneal cavity [15].

Although numerous reviews investigate various aspects of systemic chemotherapy [5,7,9,10] and IPC chemotherapy in gastric cancer [13,14,15], there is a lack of comprehensive reviews focusing specifically on these treatment modalities in the context of locally advanced gastric cancer (LAGC). To address this gap, the present review aims to provide an overview of the current state of knowledge regarding perioperative systemic chemotherapy, including its combination with targeted therapy, as well as the two major IPC therapies—HIPEC and PIPAC—in the management of resectable LAGC. Special attention is given to identifying existing controversies and knowledge gaps while proposing directions for future research.

## 2. Methodology of Study Search and Selection

To achieve the review’s objectives, a comprehensive search of the literature available in evidence-based medicine databases was conducted. The primary databases used for completed studies included MEDLINE/PubMed, Web of Science, and ScienceDirect, chosen for their extensive coverage of peer-reviewed publications in oncology and cancer care. For ongoing clinical trials, ClinicalTrials.gov served as the principal source due to its robust repository of trial data. By leveraging these platforms, the search aimed to ensure a comprehensive review of both completed studies and ongoing research efforts, providing a holistic understanding of current and emerging chemotherapeutic strategies for LAGC.

The search strategy was designed to identify studies and trials relevant to the review’s scope. For completed studies, the following key terms and combinations were used: “gastric cancer” OR “gastric cancer, locally advanced” AND “chemotherapy” OR “chemotherapy, adjuvant” OR “chemotherapy, neoadjuvant” OR “chemotherapy, combination” AND “targeted therapies, molecular” AND “cancer chemotherapy, regional perfusion.” In MEDLINE/PubMed, the Medical Subject Headings (MeSH) database was referenced to validate the key terms, ensuring the inclusion of all relevant synonyms and variations for precise and comprehensive retrieval of records. For searches conducted in ClinicalTrials.gov, the following parameters were applied: condition/disease: “gastric cancer” OR “gastric adenocarcinoma” OR “gastric carcinoma” OR “locally advanced gastric cancer”, OR “locally advanced gastric carcinoma”, OR “locally advanced gastric adenocarcinoma”, and intervention/treatment: “chemotherapy”, “systemic chemotherapy”, “targeted therapy”, AND “intraperitoneal chemotherapy”, “HIPEC”, OR “PIPAC”. These parameters were tailored to capture ongoing trials focusing on systemic and regional chemotherapy approaches in treating LAGC.

All relevant information was critically reviewed for its applicability to the setting of resectable LAGC, with studies focusing exclusively on non-resectable disease being excluded. The primary data extracted from the identified scientific evidence were systematically analyzed using an inductive approach, then summarized and categorized into significant themes aligned with the scope of the review. These themes included standard perioperative chemotherapy regimens, combination chemotherapy and targeted therapy regimens, and IPC, including HIPEC and PIPAC. The thematic analysis facilitated the synthesis of key findings, highlighting the current state of knowledge and ongoing research regarding chemotherapy for LAGC. Particular emphasis was placed on comparing the outcomes of various systemic and IPC modalities, evaluating their effectiveness, safety profiles, and integration into multimodal treatment strategies.

## 3. Classification and Staging Systems for Gastric Cancer

Several classification systems are used for gastric cancer, each based on a distinct approach. The TNM system is widely employed for staging gastric cancer, providing information about tumor size (T), lymph node involvement (N), and the presence of distant metastasis (M). Histological classification systems focus on identifying and categorizing the structural and cellular characteristics of the tumor, while molecular classification systems emphasize molecular and genetic profiling to identify distinct subtypes of gastric cancer, which can guide therapeutic strategies. A comprehensive understanding of these classification and staging systems is critical for determining the role of various therapeutic agents in LAGC.

### 3.1. Histological Classification Systems

The fifth edition of the WHO classification of digestive tumors, developed in 2019, represents the most detailed pathophysiological classification of gastric cancer to date. According to this classification, gastric tumors are categorized into adenocarcinomas, other epithelial tumors, neuroendocrine tumors, stromal and mesenchymal tumors, and lymphomas. Each histological entity is further subdivided into specific subtypes [16]. While the WHO classification provides a comprehensive framework, it is frequently criticized for its complexity, particularly because many of the tumors listed are exceedingly rare. Also, the system has been questioned regarding its clinical utility, specifically its ability to reliably predict patient prognosis or response to therapy [3].

The Lauren classification, introduced in 1965, remains the most widely used histological classification system for gastric adenocarcinoma. It divides gastric cancer into two main types: intestinal and diffuse [17]. An indeterminate type was later added to account for tumors with uncommon histological features [18]. The intestinal type is the most common and is characterized by the formation of glandular structures resembling intestinal epithelium. In contrast, the diffuse type is defined by poorly cohesive cancer cells, often including signet-ring cells [17]. Intestinal-type gastric cancer is frequently associated with environmental factors and is more common in older patients, whereas diffuse-type gastric cancer is often linked to genetic predispositions and occurs more frequently in younger patients [19].

The Nakamura classification system, developed in 1968, divides gastric adenocarcinoma into differentiated, undifferentiated, and unclassified types [20]. This classification system serves as a basis for defining indications for the endoscopic resection of early gastric cancer [3].

The macroscopic classification of gastric cancer refers to the categorization of gastric tumors based on their gross appearance, which can be observed during endoscopy or surgery. Several systems have been developed to classify the macroscopic features of gastric cancer, with the Japanese Classification of Gastric Carcinoma being one of the most widely used. This system divides gastric cancer into six major subtypes based on tumor shape and characteristics. Type 0 (superficial tumor) is analogous to T1 tumors. Type I (mass) is described as a tumor that raises above the surrounding mucosa and has a polypoid shape. Type II tumors (ulcerative) are described as an ulcerated tumor with raised margins, surrounded by a thickened gastric wall with clear margins. Type III tumors (infiltrative ulcerative) are identical to type II but without clear margins. Type IV tumors (diffuse infiltrative) are described as tumors that exhibit diffuse infiltration without marked ulceration or raised margins, while type V (unclassified tumors) refers to tumors that do not fit into the other categories or show mixed features of different types [21].

The Bormann classification system is particularly useful in describing advanced gastric cancer. It divides gastric cancer into four types, each with distinct features that can influence the treatment approach and prognosis: Type I (polypoid tumor) refers to a tumor with a broad base that is raised above the surrounding mucosa, forming a polypoid mass with no ulceration. Type II (superficial ulcer) is a tumor with a central crater or depression surrounded by raised, firm margins. Type III (excavated) refers to a more diffuse and infiltrative ulcer with irregular borders and no distinct margins, while Type IV (linitis plastica) presents as a diffusely infiltrative thickening of the gastric wall [22]. The Bormann classification system is valuable for determining the clinical management of gastric cancer, particularly for assessing the extent of the tumor and planning appropriate treatment strategies [3].

### 3.2. Molecular Classification Systems

The advances in molecular and genetic research observed during the past decades have led to the development of molecular or genomic classification systems for gastric cancer. The two most commonly used classification systems are the Cancer Genome Atlas (TCGA) and the Asian Cancer Research Group (ACRG).

The TCGA classification system was developed in 2014 following the publication of the results from comprehensive integrated genome-wide analyses of DNA copy number alterations, mutations, mRNA, miRNA, and protein expression patterns [23]. The TCGA classification delineates four distinct subtypes of gastric cancer: Epstein–Barr virus (EBV)-positive tumors, microsatellite instability (MSI) tumors, genomically stable (GS) tumors, and chromosomal instability (CIN) tumors [24]. The EBV-positive tumors are characterized by frequent *PIK3CA* mutations, DNA hypermethylation, and the amplification of genes such as Janus Kinase 2 (*JAK2)* and Programmed Death-Ligands 1 and 2 *(PD-L1/PD-L2*). These tumors also exhibit a strong immune response attributed to the presence of the EBV [25]. The MSI tumors are marked by a high mutation burden and substantial hypermethylation, particularly of mismatch repair genes [26]. The GS tumors, commonly associated with diffuse-type gastric cancer, exhibit a lack of significant chromosomal instability. Alterations in the *RHOA* gene and the *CLDN18-ARHGAP* fusion are frequently observed in this subtype [27]. The CIN tumors, predominantly linked to intestinal-type gastric cancer, display extensive chromosomal instability. These tumors often involve amplifications of genes such as *ERBB2*, *VEGFA*, and *MET* and are generally associated with poor differentiation and a more aggressive clinical course [28].

The ACRG classification system was developed in 2015 based on mRNA expression profiling. This classification shows some overlap with the TCGA classification system, distinguishing four molecular subtypes of gastric cancer: MSI, microsatellite-stable (MSS)/epithelial–mesenchymal transition, MSS/tumor protein 53 (TP53)-active, and MSS/TP53-inactive [29]. The MSI subtype in the ACRG classification is similar to that identified in the TCGA system; it is characterized by a high mutational burden and instability in microsatellite regions. It can be identified by the expression of MutL protein homolog 1 [30]. The MSS/epithelial–mesenchymal transition (EMT) subtype involves a shift from epithelial to mesenchymal phenotypes, often associated with increased invasion and metastasis. These tumors typically exhibit high levels of EMT markers and are generally more resistant to conventional therapies [31]. The MSS/TP53-active subtype is characterized by the activation of the *TP53* tumor suppressor gene, which is involved in regulating cell cycle progression and apoptosis [32].

The molecular classification systems guide diagnostic and therapeutic decision-making for LAGC and provide prognostic insights. For example, studies have shown that MSI is generally associated with a better prognosis compared to MSS tumors and typically demonstrates favorable responses to immune checkpoint inhibitors (ICIs). However, the effectiveness of standard chemotherapy for MSI tumors remains controversial [26]. The MSS/TP53-active subtype is frequently associated with better differentiation and lower aggressiveness compared to other gastric cancer subtypes [32]. Similar to MSI tumors, EBV-positive gastric cancers generally have a more favorable prognosis. Given their high *PD-L1* expression and immune infiltration, EBV-associated gastric cancers have shown promising responses to ICIs [25]. In contrast, the MSS/TP53-inactive subtype is characterized by a loss of *TP53* activity, leading to genomic instability and an increased potential for tumor progression and metastasis. These tumors often exhibit poor differentiation and are linked to a more aggressive clinical course [33].

Although the TCGA and ACRG classification systems provide a deeper understanding of tumor biology, robust evidence supporting their prognostic and therapeutic implications exists primarily for the MSS/TP53 [32], MSI [34], and EBV-positive [35] gastric cancer subtypes, based on biomarker analyses and clinical studies. The clinical significance of other molecular subtypes, particularly in terms of prognosis and treatment selection, remains an active area of investigation, with ongoing research exploring their therapeutic relevance and potential for targeted treatment strategies [36]. Currently, molecular subgroup testing is not yet a standard component of clinical practice, and the histological Lauren classification remains the most widely used system in clinical trials for gastric cancer [3].

### 3.3. TNM Classification System

The TNM classification system, developed by the American Joint Committee on Cancer and the Union for International Cancer Control, is the most widely used staging system for gastric cancer. The eighth edition, introduced in 2017, offers refined staging criteria based on clinical and pathological findings. The primary tumor (T) is staged according to its depth of invasion: Tis (carcinoma in situ), T1 (invasion of lamina propria, muscularis mucosae, or submucosa), T2 (invasion of muscularis propria), T3 (penetration into subserosal connective tissue without reaching the visceral peritoneum or adjacent structures), and T4 (invasion of the visceral peritoneum or adjacent organs/structures). Lymph node involvement (N) is categorized as N0 (no regional lymph node involvement), N1 (1–2 affected nodes), N2 (3–6 affected nodes), N3a (7–15 affected nodes), and N3b (16 or more affected nodes). Distant metastasis (M) is classified as M0 (no distant metastasis) or M1 (presence of distant metastasis) [37].

These factors are combined into stages 0-IV: stage 0 (early-stage disease, carcinoma in situ (Tis, N0, M0)), stage I (localized disease, limited invasion into the gastric wall (T1-T2, limited nodal involvement)), stage II (deeper invasion into the gastric wall, with regional lymph node involvement), stage III (advanced local disease with significant nodal involvement or penetration into adjacent structures), and stage IV (presence of distant metastasis (M1)) [37]. Diagnostic imaging is most commonly used to enable pre-surgical staging of gastric cancer, with computed tomography and magnetic resonance imaging being the most viable approaches [38].

LAGC refers to tumors that have significantly invaded the gastric wall and/or exhibit extensive regional lymph node involvement, yet have not metastasized to distant organs. In terms of the TNM classification, resectable LAGC generally describes tumors that have penetrated the subserosal connective tissue (T3) or invaded the serosa (visceral peritoneum) without extending to adjacent structures (T4a), with any regional lymph node status (N0–N3) and no evidence of distant metastases (M0) [39]. Clinically, LAGC is often defined as stage T2 and beyond, with or without confirmed regional nodal involvement [40]. It is important to distinguish LAGC from advanced gastric cancer, which encompasses both locally advanced disease involving adjacent structures (T4b) and distant metastasis (M1). This distinction is critical, as it carries significant implications for treatment strategies and prognosis [41].

## 4. Multimodal Approach to Treatment of Locally Advanced Gastric Cancer

To select the optimal treatment option for an individual patient with LAGC, a variety of factors must be evaluated before treatment planning. Patient-related factors include age, general health status, and expected treatment outcomes. Tumor-related factors encompass the clinical stage based on the TNM classification system and the molecular characteristics of the tumor [40]. The diagnostic workup typically includes upper endoscopy with biopsy and contrast-enhanced computed tomography scanning of the abdominal, pelvic, and thoracic cavities. These assessments provide critical information on the tumor’s morphology, histology, and genetic profile, as well as the extent of gastric wall invasion (T), lymph node involvement (N), and the presence of distant metastases (M), including their locations, if present. Additional diagnostic modalities, such as upper endoscopic ultrasound and diagnostic laparoscopy with or without peritoneal washings, are valuable for distinguishing between early gastric cancer and LAGC [42]. The inclusion of diagnostic laparoscopy in the routine diagnostic workup for LAGC is justified by evidence showing that up to 52% of patients may have peritoneal metastases that are undetectable in imaging studies [40].

Radical surgery with adequate gastric resection and lymphadenectomy is the preferred surgical technique for LAGC. The extent of stomach resection is guided by tumor location and histological type. Large tumors or those located along the lesser curvature of the stomach are best managed with total gastrectomy. For ≥T2 tumors with an expansive growth pattern, distal or subtotal gastrectomy may be performed, ensuring a proximal margin of at least 3 cm. In contrast, ≥T2 tumors with an infiltrative growth pattern require a proximal margin of at least 5 cm to minimize the risk of residual disease [40,42].

The extent of lymphadenectomy during radical surgery is a critical consideration. Currently, D2 resection is regarded as the gold standard surgical procedure for LAGC. D2 resection involves the removal of perigastric lymph nodes (stations 1–6) along the lesser and greater curvatures, as well as second-tier lymph nodes located along major blood vessels, including the celiac artery, splenic artery, common hepatic artery, and left gastric artery (stations 7–11) [40]. D2 resection is recommended by numerous international clinical practice guidelines, including the National Comprehensive Cancer Network Clinical Practice Guidelines in Gastric Cancer [42], the Japanese Gastric Cancer Association Treatment Guidelines [43], and the Italian Research Group for Gastric Cancer Guidelines [44]. Its efficacy is supported by evidence from large-scale prospective clinical trials, further solidifying its role in the management of LAGC [45].

However, D2 resection alone does not provide a cure for LAGC. A multimodal treatment strategy, including systemic chemotherapy, potentially combined with targeted therapy and IPC, is recommended to optimize treatment outcomes in LAGC [40].

## 5. Chemotherapy Regimens for Locally Advanced Gastric Cancer

Perioperative chemotherapy has become the standard for the management of LAGC. It typically begins in the preoperative period, with a few cycles of neoadjuvant chemotherapy, and extends into the postoperative period. A variety of regimens are used for perioperative chemotherapy in LAGC, involving different combinations of cytotoxic agents. Most regimens can be classified into two broad categories: doublet regimens (a combination of two chemotherapeutic agents) and triplet regimens (a combination of three chemotherapeutic agents). In general, triplet regimens are considered more effective in achieving tumor control and improving survival outcomes but are associated with a higher rate of side effects. Doublet regimens, on the other hand, demonstrate lower efficacy but have a more favorable toxicity profile, making them more suitable for patients who cannot tolerate the increased toxicity associated with triplet regimens [40].

The ECF regimen, first evaluated in the MAGIC trial published in 2006, compared the effectiveness of perioperative chemotherapy (three preoperative and three postoperative cycles of 50 mg/m^2^ epirubicin, 60 mg/m^2^ cisplatin on day one, and a continuous intravenous infusion of 200 mg/m^2^ 5-fluorouracil over 21 days) combined with surgery versus surgery alone. Patients receiving the ECF regimen in addition to surgery demonstrated significantly improved outcomes compared with those undergoing surgery alone. These included a higher likelihood of overall survival (hazard ratio (HR) 0.75; 95% confidence interval (CI), 0.60–0.93), a higher five-year survival rate (36% vs. 23%, respectively), and improved progression-free survival [46]. Prior to the introduction of the FLOT regimen, the ECF regimen was regarded as a cornerstone in the treatment of LAGC.

The FLOT regimen gained prominence following the publication of the FLOT4-AIO trial results in 2019, which compared the ECF regimen (administered in three preoperative and three postoperative cycles) with the FLOT regimen (four preoperative and four postoperative two-week cycles consisting of 50 mg/m^2^ docetaxel, 85 mg/m^2^ oxaliplatin, 200 mg/m^2^ leucovorin, and 2600 mg/m^2^ 5-fluorouracil as a 24 h infusion on day one). The FLOT regimen demonstrated superior outcomes, including a higher overall survival (HR 0.77; 95% CI 0.63–0.94) and a longer median overall survival (50 months vs. 35 months). Both regimens were comparable in terms of the rates of serious adverse events and the number of toxic deaths [47]. Other studies have also supported the efficacy of the FLOT regimen [48,49,50]. In addition, the FLOTA regimen was proposed, which combines the traditional FLOT regimen with apatinib, a tyrosine kinase inhibitor, administered preoperatively. The FLOTA regimen demonstrated improved perioperative efficacy compared to FLOT, including a higher objective response rate (80.65% vs. 50.00%) and a greater reduction in target lesion size. However, patients receiving FLOTA experienced a significantly higher rate of preoperative adverse events, such as diarrhea, pain, oral mucositis, and hand-foot syndrome, compared with those receiving FLOT [51]. Currently, the FLOT regimen is widely established as the preferred standard for perioperative chemotherapy in the treatment of LAGC in many clinical centers worldwide [3].

The XELOX regimen, also interchangeably called CAPOX, is a combination of capecitabine and oxaliplatin and is commonly used for both neoadjuvant and adjuvant chemotherapy in the treatment of LAGC. The regimen offers a simplified alternative to more traditional 5-fluorouracil-based regimens, such as ECF or FLOT. The effectiveness of the XELOX regimen was first evaluated in the CLASSIC trial, which randomized patients to either eight 3-week postoperative cycles of 1000 mg/m^2^ capecitabine and 130 mg/m^2^ oxaliplatin or surgery alone. The study found improved 3-year disease-free survival in the XELOX group compared to the surgery-alone group (74% vs. 59%), although the XELOX group experienced higher rates of grade 3 or 4 adverse events (56% vs. 6%) [52]. The XELOX regimen is particularly indicated for patients who are not eligible for or do not tolerate more aggressive regimens. The ongoing PECORINO trial is aimed at evaluating the rates of chemotherapy-related adverse effects and intolerance in patients with resectable gastric cancer receiving either neoadjuvant FLOT or XELOX [53]. However, a retrospective study reported the inferiority of XELOX to FLOT as a neoadjuvant chemotherapy regimen for LAGC [54], and another study demonstrated the inferiority of XELOX to DOX in terms of pathological complete response rate (pCRR) and 3-year overall survival [55]. These findings raise concerns regarding the effectiveness of XELOX compared to triplet neoadjuvant chemotherapy regimens for LAGC.

The FOLFOX regimen (a combination of leucovorin, 5-fluorouracil, and oxaliplatin) is another triplet chemotherapy regimen used for the treatment of LAGC. It is employed both as an adjuvant and neoadjuvant therapy and was initially proposed for patients with advanced gastric cancer. Multiple clinical trials have investigated the use of the FOLFOX regimen and its modifications in the perioperative treatment of gastric cancer [40]. A study by Li et al. compared the effectiveness of perioperative FOLFOX versus postoperative FOLFOX in patients with LAGC. The 4-year overall survival was 78% in the neoadjuvant arm compared to 51% in the adjuvant arm, with no treatment-related deaths reported in either arm [56]. The FOLFOX4 regimen consists of 85 mg/m^2^ oxaliplatin, 200 mg/m^2^ leucovorin, and 5-FU at 400 mg/m^2^ as a bolus, followed by a continuous infusion of 600 mg/m^2^ for 22 h on days one and two. A retrospective study comparing the effectiveness of the FOLFOX4 regimen as preoperative chemotherapy for advanced gastric cancer concluded that it improved patient survival without increasing adverse events [57]. The modified FOLFOX6 regimen, consisting of 85 mg/m^2^ oxaliplatin, 400 mg/m^2^ leucovorin, and 5-FU at 400 mg/m^2^ as a bolus, followed by a continuous infusion of 2400 mg/m^2^ for 46 h on day one, administered over three cycles, was reported to be both well tolerated and effective [58]. In general, the advantages of FOLFOX as a perioperative regimen for LAGC include its relatively mild side-effect profile, making it a suitable option for patients who may not tolerate more intensive treatments. Yet, real-world studies indicate that FOLFOX is inferior to FLOT in terms of patient outcomes [3].

The DOX regimen (60 mg/m^2^ docetaxel, 130 mg/m^2^ oxaliplatin, and 1000 mg/m^2^ capecitabine) has gained less popularity than FLOT or FOLFOX as the perioperative treatment of LAGC. Nevertheless, several studies have evaluated its safety and effectiveness compared to other systemic chemotherapy regimens. Tian et al. compared the DOX regimen with XELOX. The DOX regimen demonstrated higher pCRR (16.1% vs. 4.3%), 3-year overall survival (56.9% vs. 44.6%), and 3-year disease-free survival (45.2% vs. 40.2%) compared to the XELOX regimen in patients with potentially resectable advanced gastric cancer [55]. When DOX is compared with FLOT, comparable radiological outcomes were reported in terms of disease response, progression, and stability [59]. The DOX regimen may be considered a viable alternative to 5-fluorouracil-based regimens, particularly for patients who may not tolerate prolonged infusions of 5-fluorouracil. However, phase III clinical trials are needed to further validate its efficacy and long-term outcomes, as well as to establish standardized protocols for its use in the neoadjuvant setting [60].

The DOS regimen combines 60 mg/m^2^ docetaxel, 100 mg/m^2^ oxaliplatin, and 40–60 mg/m^2^ S-1 (depending on body surface area). The DOS regimen offers advantages over traditional perioperative chemotherapy regimens through the inclusion of S-1 instead of 5-fluorouracil, eliminating the need for continuous intravenous infusions and minimizing associated side effects. The MATCH trial compared the effectiveness of the DOS regimen with the SOX regimen (S-1 plus oxaliplatin) in LAGC. Compared to SOX, DOS significantly improved the major pathological response rate (≤10% residual viable tumor) (25.4% vs. 11.8%) and tended to result in longer 3-year progression-free survival (78.9% vs. 61.8%) and 3-year overall survival (57.5% vs. 49.2%) [61]. The PRODIGY trial evaluated the DOS regimen in both neoadjuvant and adjuvant formats compared with adjuvant S-1 monotherapy. Results indicated that 3-year progression-free survival was significantly higher in the DOS group, with toxicity levels below the expected rates [62]. It is important to note that S-1 (oral tegafur, gimeracil, and oteracil) is a drug commonly used in East Asian countries (Japan, China, and South Korea), where S-1-containing regimens have gained substantial popularity. In contrast, FLOT remains the preferred regimen in many European countries. Although DOS appears to be a promising neoadjuvant chemotherapy regimen for LAGC, further large-scale phase III clinical trials are needed to establish its efficacy and safety compared to globally accepted standards such as FLOT. Additionally, its adoption outside East Asian regions may be limited by the availability and familiarity with S-1-based therapies [63].

The SOX regimen (S-1 and oxaliplatin) is also used in both neoadjuvant and adjuvant settings. Similar to the DOS regimen, it is more popular in East Asian countries, where S-1 is widely available and well studied. The regimen is typically administered in 3-week cycles, with S-1 taken twice daily for two weeks, followed by a 1-week rest period. In the MATCH trial, SOX was compared with the DOS regimen in patients with LAGC. The DOS regimen demonstrated better pathological response and showed a trend toward longer 3-year progression-free survival and 3-year overall survival, with a comparable safety profile to the SOX regimen [61]. The Dragon III trial compared the SOX and FLOT regimens in a neoadjuvant setting. Although the study found that the SOX regimen resulted in a higher rate of complete or subtotal tumor regression (32.4% vs. 20.0%), this difference was not statistically significant. However, the study reported comparable toxicity profiles for the SOX and FLOT regimens [64]. Several ongoing clinical trials are evaluating variations of the SOX regimen and its combinations with other therapeutic agents to further optimize its efficacy and safety profile.

The DCF perioperative regimen (75 mg/m^2^ docetaxel, 75 mg/m^2^ cisplatin, and 750 mg/m^2^/day 5-fluorouracil) is typically administered in three neoadjuvant and three adjuvant cycles. Ferri et al. reported that a metabolic response to chemotherapy was achieved in 76% of patients with LAGC receiving DCF, and resection was possible in 95.3% of cases, with a pCRR of 9.75% [65]. A comparison between the DCF regimen and FLOT demonstrated the inferiority of DCF in terms of pCRR and disease-free survival, while showing comparable overall survival [66]. Given the availability of multiple studies comparing DCF with FOLFOX, a meta-analysis was conducted, which revealed comparable effectiveness between the two regimens. However, FOLFOX was superior regarding the frequency of side effects such as nausea and vomiting, anemia, thrombocytopenia, and leukopenia, whereas DCF showed lower rates of sensory neurotoxicity [67]. In general, the DCF regimen is associated with poor tolerability, which has led to the development of modified DCF protocols. For example, the modified DCF regimen (40 mg/m^2^ docetaxel, 40 mg/m^2^ cisplatin, and 400 mg/m^2^ 5-fluorouracil) was compared with the EOX regimen (epirubicin, oxaliplatin, and capecitabine), demonstrating comparable effectiveness and toxicity profiles [68]. A meta-analysis evaluating the effectiveness and safety of various modified DCF regimens concluded that they exhibit an adequate overall performance, balancing efficacy and tolerability [69].

Kalachand et al. reported the use of the EOX regimen in the perioperative setting. Preoperative EOX treatment enabled 85% of patients with LAGC to proceed to surgery, and 67% of them achieved R0 resection (a complete surgical removal of the tumor with negative microscopic margins). The pCRR was 6%, and grade 3–4 toxicities were observed in 17% of patients [70]. When compared to XELOX (capecitabine plus oxaliplatin), EOX demonstrates similar effectiveness in terms of disease-free survival and overall survival, but has an inferior toxicity profile due to a higher incidence of leukopenia, neutropenia, fatigue, and vomiting [71]. The modified FLOT regimen (docetaxel, oxaliplatin, leucovorin, and 5-fluorouracil administered in 2-week cycles) demonstrated superior effectiveness over EOX in terms of 3-year event-free survival but exhibited inferior safety, with higher rates of grade 3 and 4 neutropenia and febrile neutropenia [72]. When compared with DCF, EOX showed comparable effectiveness but a superior safety profile [68]. In comparison with FOLFOX (leucovorin, 5-fluorouracil, and oxaliplatin), the EOX regimen resulted in fewer patients presenting with deeply invasive and metastatic cancer at follow-up and a higher number of patients achieving tumor regression grades 3 and 4 [73]. Overall, EOX is considered a valuable treatment option for gastric cancer, particularly for patients with locally advanced disease or those requiring a more tolerable alternative to more aggressive chemotherapies such as DCF or FLOT. Table 1 summarizes the most common perioperative chemotherapy regimens for LAGC.

The appearance of targeted therapy has changed the treatment landscape for LAGC by offering more personalized approaches that specifically target the molecular pathways involved in tumor growth and progression. A variety of combination chemotherapy and targeted therapy regimens exist, aiming to enhance the treatment efficacy for LAGC.

## 6. Combination Chemotherapy and Targeted Therapy Regimens for Locally Advanced Gastric Cancer

Over the past decade, scientific research has focused extensively on the development and evaluation of novel targeted therapy agents for the treatment of gastric cancer, particularly advanced gastric cancer, in combination with standard chemotherapy regimens. The targeted therapy agents evaluated include monoclonal antibodies directed at human epidermal growth factor receptor 2 (HER2), vascular endothelial growth factor (VEGF), hepatocyte growth factor/c-MET (mesenchymal–epithelial transition factor), mammalian target of rapamycin (mTOR), fibroblast growth factor receptor (FGFR), and tissue factor signaling pathways, among others. These agents aim to enhance treatment efficacy by specifically targeting the molecular pathways critical to tumor growth, angiogenesis, and metastasis. However, outside the context of advanced gastric cancer, their effectiveness in LAGC remains less well understood, as relatively few trials have been completed to date, although many are currently ongoing [40].

### 6.1. Combination Chemotherapy and HER2 Inhibitor Regimens

Approximately 20% of gastric cancers exhibit overexpression of the HER2 receptor, which can be targeted by anti-HER2 antibodies, such as trastuzumab [40]. The first trial investigating the benefits of adding trastuzumab to chemotherapy compared to chemotherapy alone was the ToGA trial, conducted in patients with advanced gastric cancer [74]. Since then, trastuzumab in combination with chemotherapy has become the standard first-line treatment for this category of patients. To date, there is a paucity of trials investigating the effects of trastuzumab combined with chemotherapy in patients with LAGC. He et al. evaluated the addition of trastuzumab to standard chemotherapy in both neoadjuvant and adjuvant settings. In the adjuvant setting, the combination of trastuzumab and standard chemotherapy resulted in improved overall survival compared to chemotherapy alone. In the neoadjuvant setting, trastuzumab addition led to enhanced tumor pathological regression and downstaging compared to chemotherapy alone [75].

The efficacy of trastuzumab in combination with atezolizumab, an ICI and the XELOX regimen was evaluated against trastuzumab and XELOX. Patients with HER2-positive LAGC received three preoperative and five postoperative cycles of 1200 mg atezolizumab, 6 mg/kg trastuzumab, 130 mg/m^2^ oxaliplatin, and 1000 mg/m^2^ capecitabine. The trial demonstrated that the combination of atezolizumab, trastuzumab, and XELOX resulted in a higher pCRR compared to trastuzumab and XELOX (38.1% vs. 14.3%) [76]. The combination of trastuzumab and FLOT was also investigated in both neoadjuvant and adjuvant settings for HER2-positive LAGC. The reported R0 resection rate was 93.3%, with a pCRR of 22.2% [77]. The ongoing INNOVATION trial aims to evaluate the effectiveness of chemotherapy alone versus chemotherapy plus trastuzumab versus chemotherapy plus trastuzumab and pertuzumab in the perioperative treatment of HER2-positive localized gastric cancer [78]. As the trial is still in progress, no results are currently available. One reason for the limited investigation of trastuzumab in LAGC is the concern over its safety profile, as it has been associated with severe adverse effects, including myelosuppression and interstitial lung disease [79]. Further studies are needed to validate its efficacy and safety in this setting.

Disitamab, another HER2-targeted agent, has also been evaluated in combination with chemotherapy in patients with LAGC. Chai et al. investigated the efficacy and safety of disitamab in combination with camrelizumab and S-1 in the neoadjuvant setting with three cycles of treatment. The reported R0 resection rate was 100%, and the pCRR was 31.25%. A decreased neutrophil count was the most common grade 3 adverse event, observed in 10% of patients [80]. Shen et al. described the protocol of a trial designed to evaluate the efficacy and safety of disitamab in combination with toripalimab and chemotherapy (XELOX regimen) compared to toripalimab/trastuzumab and chemotherapy (XELOX) in patients with LAGC or metastatic gastric cancer. The primary endpoint of the trial is progression-free survival [81].

### 6.2. Combination Chemotherapy and Immune Checkpoint Inhibitors Regimens

ICIs represent a rapidly advancing class of immunotherapy agents that enhance the immune system’s ability to recognize and eliminate cancer cells. These agents have garnered significant attention in recent years, with numerous studies exploring their use in combination with standard chemotherapy regimens to improve therapeutic outcomes. ICIs function by targeting specific immune checkpoint molecules, such as those expressed on immune cells or tumor cells, thereby reversing the immunosuppressive tumor microenvironment and restoring antitumor immunity. Currently, available ICIs for gastric cancer include inhibitors of PD-1 and its ligand (PD-L1), which block the interaction that typically suppresses T-cell activity, as well as inhibitors targeting cytotoxic T-lymphocyte-associated antigen 4 (CTLA-4). Additionally, emerging therapies targeting lymphocyte activation gene-3 (*LAG-3*) are under investigation, expanding the potential applications of ICIs in the treatment of LAGC [40].

Camrelizumab, in combination with apatinib and chemotherapy, was evaluated in the neoadjuvant setting for LAGC compared with chemotherapy alone. The study demonstrated the superiority of the combined treatment over chemotherapy alone in terms of major pathological response rate (33.3% vs. 17.0%), objective response rate (66.0% vs. 43.4%), and R0 resection rate (94.1% vs. 81.1%). However, the combined treatment also showed a higher rate of grade 3–4 adverse events (33.3% vs. 26.4%) [82]. Another study assessed the efficacy and safety of camrelizumab combined with the SOX regimen in the perioperative setting. The reported pCRR was 10.3%, the major pathological response rate was 9.0%, and R0 resection was achieved in 96.6% of cases. Treatment-emergent adverse events of any grade were observed in 82.8% of patients [83]. Also, a study evaluated the efficacy and safety of neoadjuvant camrelizumab, rivoceranib, and the SOX chemotherapy regimen compared with the SOX regimen alone in LAGC. The pCRR in the combined therapy group was 18.3% versus 5% in the SOX-alone group, while grade 3 treatment-associated adverse events occurred in 34% versus 17% of patients, respectively [84].

Atezolizumab is another ICI, the efficacy of which has been evaluated in combination with other targeted therapy agents and chemotherapy. The study by Peng et al. referenced above evaluated the efficacy of atezolizumab in combination with trastuzumab and the XELOX regimen, compared to trastuzumab and XELOX alone. The combination of atezolizumab, trastuzumab, and XELOX resulted in a higher pCRR compared to trastuzumab and XELOX (38.1% vs. 14.3%) [76]. The PANDA trial examined the efficacy and safety of a combined atezolizumab and DOX chemotherapy regimen administered in a neoadjuvant setting. A major pathological response was reported in 70% of patients, while the pCRR was 45% [85]. The ongoing DANTE trial is designed to study the efficacy and safety of atezolizumab combined with FLOT chemotherapy versus FLOT alone in patients with LAGC exhibiting high immune responsiveness. The primary endpoint is event-free survival, while the main secondary endpoints include pCRR, overall survival, R0 resection rate, and safety/tolerability characteristics [86].

Pembrolizumab is also an ICI that has been evaluated for the treatment of LAGC in combination with chemotherapy. The KEYNOTE-585 study assessed the antitumor activity of pembrolizumab administered in three preoperative and three postoperative cycles in combination with perioperative chemotherapy (a cisplatin-based doublet regimen or FLOT) compared to chemotherapy alone. The combination of pembrolizumab with chemotherapy resulted in a higher pCRR than chemotherapy alone (12.9% vs. 2.0%), longer event-free survival (44.4 months vs. 25.3 months), and improved median overall survival (60.7 months vs. 58.0 months). However, the rate of grade 3–4 adverse effects was also higher in the pembrolizumab group (78% vs. 74%) [87]. Kennedy et al. investigated the benefits of perioperative pembrolizumab in MSI, EBV-positive, or PD-L1-positive surgically resectable tumors. All patients received two cycles of preoperative pembrolizumab followed by surgery and adjuvant capecitabine plus pembrolizumab. Tumor downstaging was observed in 50% of patients, and the pCRR was 9.1% [88].

The clinical benefits of adding toripalimab to a perioperative chemotherapy regimen in patients with LAGC were investigated by Yuan et al. All patients were randomized into one of two groups: the first group received three preoperative and five postoperative cycles of SOX/XELOX, while the second group received toripalimab in combination with SOX/XELOX. The trial demonstrated the superiority of the combined treatment over chemotherapy alone in terms of improved pCRR (22.2% vs. 7.4%) and reduced surgical morbidity (11.8% vs. 13.5%). However, the rate of grade 3–4 adverse effects was higher in the combination therapy group (35.2% vs. 29.6%) [89]. Another study reported that the combination of toripalimab with XELOX in a perioperative setting (4 preoperative and 4 postoperative cycles) resulted in a 100% R0 resection rate, a 92.9% major pathological response rate, and a 78.6% pCRR [90].

The combination of perioperative nivolumab with the SOX regimen achieved a pCRR of 28.26% and a major pathologic response rate of 41.3% in a cohort of patients with LAGC [91]. A small case series reported the benefits associated with adding nivolumab to an oxaliplatin-based chemotherapy regimen in LAGC patients, including tumor downstaging, which facilitated subsequent radical surgery [92].

Sintilimab is being evaluated in a variety of ongoing studies in combination with chemotherapy regimens for patients with LAGC. The ongoing TACTIC trial (NCT05593458) is designed to compare the efficacy and safety of transarterial neoadjuvant chemotherapy combined with SOX plus sintilimab versus traditional intravenous chemotherapy with SOX plus sintilimab. The study endpoints include the major pathological response rate, pCRR, 2-year overall survival, 2-year disease-free survival rates, R0 resection rates, and adverse events [93]. Another ongoing study (NCT06459921) aims to compare the efficacy and safety of the SOX regimen plus sintilimab with the FLOT regimen plus sintilimab. The primary endpoint is a R0 resection rate, while disease-free survival and the adverse event rate are secondary endpoints [94]. In addition, the efficacy and safety of neoadjuvant sintilimab in combination with XELOX are being evaluated in another study (NCT04065282). The primary endpoint is the pCRR, while secondary endpoints include the objective response rate, tumor regression rate, disease-free survival, and overall survival rate [95]. Since all of these studies are still recruiting patients, no results have been reported thus far.

### 6.3. Combination Chemotherapy and VEGF Inhibitor Regimens

Inhibitors of VEGF are a class of targeted therapies that disrupt angiogenesis by binding to its receptors (VEGFR) on endothelial cells. Anti-VEGF agents can enhance chemosensitivity due to the dual role of VEGF in the tumor microenvironment: (1) promoting aberrant and undifferentiated tumor angiogenesis and (2) exerting immunosuppressive effects on tumor cells [3]. Several inhibitors of VEGF have been investigated in combination with standard chemotherapy regimens in the context of resectable LAGC.

Apatinib was studied in combination with the FLOT regimen and compared with FLOT alone. The FLOTA regimen demonstrated improved perioperative efficacy compared to FLOT, including a higher objective response rate (80.65% vs. 50.00%) and a greater reduction in target lesion size. However, patients receiving FLOTA experienced a significantly higher rate of preoperative adverse events, such as diarrhea, pain, oral mucositis, and hand-foot syndrome, compared with those receiving FLOT [51]. Apatinib was also studied in combination with camrelizumab and S-1 plus paclitaxel in the neoadjuvant setting for LAGC, compared to S-1 plus paclitaxel alone. The combination regimen demonstrated superiority over chemotherapy alone in terms of a higher major pathological response rate (33.3% vs. 17.0%), objective response rate (66.0% vs. 43.4%), and R0 resection rate (94.1% vs. 81.1%). Nevertheless, it was associated with a higher rate of grade 3–4 adverse events (33.3% vs. 26.4%) [82]. A combination of apatinib, camrelizumab, and the SOX regimen was evaluated in the perioperative setting for LAGC, compared to SOX alone. Adding apatinib and camrelizumab to SOX resulted in a higher pCRR (18.3% vs. 5.0%), improved major pathological response rate (51.1% vs. 37.8%), and a higher R0 resection rate (98.7% vs. 94.2%). However, this combination was associated with higher rates of grade 3–4 adverse events (36.3% vs. 16.3%) [96].

The efficacy of a combined bevacizumab, docetaxel, cisplatin, and capecitabine regimen in LAGC was investigated in a neoadjuvant setting. This combination resulted in an objective response rate of 55.0% and a R0 resection rate of 90.0% [97]. Rayes et al. described the effects of adding bevacizumab to a neoadjuvant regimen of docetaxel and oxaliplatin in a sample of patients with LAGC and metastatic gastric cancer. The authors reported that a pCRR was achieved in 5% of patients, while partial responses were observed in 37% [98]. Since the sample included patients with metastatic disease, the findings of this study should be interpreted with caution. Ma et al. evaluated the value of adding bevacizumab to a neoadjuvant chemotherapy regimen consisting of docetaxel, oxaliplatin, and 5-fluorouracil compared with chemotherapy alone. After three years, the disease-free survival was higher in the group receiving bevacizumab compared to the chemotherapy-alone group (15.2 months vs. 12.3 months), as was the overall survival rate (17.6 months vs. 16.4 months) [99]. Yin and Luo reported the efficacy and safety of neoadjuvant bevacizumab combined with various chemotherapy regimens compared with chemotherapy alone in patients with LAGC. The authors observed an improved objective response rate (52.2% vs. 35.4%), disease control rate (91.3% vs. 81.3%), and surgical resection rate (95.7% vs. 85.4%), while maintaining a comparable safety profile [100].

### 6.4. Other Promising Combination Regimens

Claudin-18 isoform 2 (CLDN18.2) inhibitors target this protein to disrupt cancer cell growth or mediate immune responses against tumor cells. The overexpression of CLDN18.2 is observed in some patients with gastric cancer, referred to as CLDN18.2-positive cases. The NEO-CLAUD trial (NCT06732856) is designed to evaluate the efficacy and safety of a neoadjuvant regimen combining zolbetuximab and DOS specifically in patients with LAGC [101]. As the trial is ongoing, no results have been reported to date. Table 2 presents an overview of combination chemotherapy and targeted therapy regimens for LAGC.

## 7. Intraperitoneal Chemotherapy for Locally Advanced Gastric Cancer

It has been estimated that among all gastrointestinal tract malignancies, gastric cancer carries the highest risk of peritoneal disease [102]. Tumor cells in gastric cancer can disseminate through direct contact once full-thickness invasion of the gastric wall occurs. When the serosa is breached, even at a microscopic level, tumor cells can detach and enter the peritoneal cavity. These free-floating cells may adhere to and implant on the peritoneal lining, leading to the formation of metastatic nodules. In addition, peritoneal dissemination can occur through the lymphatic spread or transcoelomic migration of tumor cells. Surgical trauma can also facilitate dissemination, particularly when tumor cells are present at resection margins or along sites of lympho-vascular transection [3].

IPC was developed to enhance the efficacy of systemic chemotherapy while maintaining an acceptable safety profile. Depending on the clinical context, IPC can be administered with curative, prophylactic, or palliative intent. Initially designed for patients with established peritoneal carcinomatosis, IPC was predominantly utilized for palliative purposes. However, ongoing research continues to refine and optimize IPC modalities, expanding its applications to include patients with LAGC and exploring its potential in prophylactic settings. Several approaches have been investigated to improve outcomes in LAGC. One such modality is HIPEC, introduced into clinical practice in the 1980s as an adjunct to cytoreductive surgery. Another promising technique is PIPAC, first introduced in 2011 [13].

### 7.1. Hyperthermic Intraperitoneal Chemotherapy for Locally Advanced Gastric Cancer

In its classical approach, during cytoreductive surgery, all visible peritoneal lesions greater than 1–2 mm in size are removed to enable HIPEC to eliminate free-floating cancer cells and peritoneal micrometastases. Following surgery, the heated chemotherapeutic agent—typically heated to 41–43 °C—is delivered intraoperatively for 30–120 min. The selection of the chemotherapeutic agent is based on its enhanced efficacy under hyperthermic conditions, with commonly used agents including cisplatin and doxorubicin, while mitomycin C was more commonly used in earlier years [103]. The blood–peritoneal barrier plays a critical role in minimizing the systemic absorption of the chemotherapeutic agent, thereby reducing the risk of significant systemic side effects. At the same time, the elevated temperature enhances drug penetration into tumor tissues and impairs DNA repair mechanisms in cancer cells, increasing their susceptibility to the chemotherapy [104].

Although HIPEC was historically designed to treat peritoneal carcinomatosis, its potential application in patients with LAGC has only recently become a focus of research. Preoperative imaging often underestimates peritoneal involvement, and up to 52% of patients may have microscopic peritoneal metastases that are undetectable in imaging studies [40]. This highlights the potential for HIPEC to be utilized with prophylactic intent in LAGC to address occult peritoneal disease and reduce the risk of recurrence.

Yu et al. conducted a study comparing the efficacy and safety of a combination of HIPEC and systemic chemotherapy with systemic chemotherapy alone in patients with resectable LAGC. Cisplatin at a dose of 40 mg/m^2^ was used for HIPEC, while the SOX regimen was employed for systemic chemotherapy. Both modalities were administered in the adjuvant setting. The disease-free survival rate was significantly higher in the HIPEC plus chemotherapy group compared to the chemotherapy-alone group (73.8% vs. 61.2%). However, the 3-year overall survival rate was slightly higher, though not significantly so, in the chemotherapy-alone group (77.6% vs. 73.9%). No differences in the rate of adverse effects were observed between the groups [105]. Similarly, Fan et al. evaluated HIPEC with cisplatin in combination with SOX chemotherapy versus SOX alone in the adjuvant setting for resectable LAGC. The peritoneal recurrence rate was lower in the group treated with HIPEC plus SOX compared to the SOX-alone group (84.8% vs. 88.2%). The rate of adverse events was comparable between the two groups [106].

Huang et al. compared the efficacy of fibrin-sealant-delivered cisplatin chemotherapy combined with systemic cisplatin to cisplatin-based HIPEC combined with systemic cisplatin in the adjuvant setting for LAGC. The reported 3-year overall survival rate was higher in the fibrin-sealant group compared to the HIPEC group (61.9% vs. 42.8%). Additionally, grade 3 and 4 adverse events or kidney dysfunction were more common in the HIPEC group (47.6% vs. 28.6%) [107]. Reutovich et al. reported the results of a study comparing the efficacy of prophylactic HIPEC with 50 mg/m^2^ of cisplatin and doxorubicin combined with systemic chemotherapy (regimen not specified) in the adjuvant setting for LAGC to HIPEC with surgery alone. The 3-year metastasis-free survival rate was significantly higher in the group receiving combination therapy compared to the HIPEC-alone group (91.0% vs. 48.6%). Similarly, the progression-free survival rate was improved in the combination therapy group (47.1% vs. 19.6%) [108].

Two meta-analyses (both published in 2022) evaluated the pooled efficacy and safety of adjuvant HIPEC in resectable LAGC, although they reached different conclusions. The meta-analysis by Zhuang et al. reported better 1-, 3-, and 5-year overall survival rates in the HIPEC group compared to the control group (odds ratios of 5.10, 2.07, and 1.96, respectively), along with lower overall recurrence rates and peritoneal recurrence rates (odds ratios of 0.41 and 0.24, respectively). However, the authors observed more favorable outcomes in the control group regarding renal and pulmonary dysfunction complications (odds ratios of 2.44 and 6.03, respectively) [109]. In contrast, Filis et al. conducted a pooled analysis of randomized controlled trials comparing HIPEC combined with adjuvant systemic chemotherapy to adjuvant systemic chemotherapy alone in resectable LAGC. This team failed to demonstrate any overall survival or progression-free survival benefits associated with the combined chemotherapy plus HIPEC regimen compared to chemotherapy alone (relative risks of 1.11 and 0.90, respectively) [110]. The difference in conclusions regarding the benefits of prophylactic HIPEC in LAGC can largely be attributed to the inclusion criteria employed by the two meta-analysis teams, which resulted in different numbers of studies being analyzed. While Zhuang et al. included twenty-two studies overall, regardless of their design [109], Filis et al. applied stricter criteria, resulting in the inclusion of only three randomized controlled trials [110]. Both teams emphasized that the existing studies on prophylactic HIPEC in LAGC are predominantly small-scale, underlining the need for well-designed, large-scale randomized controlled trials to provide more definitive evidence on its efficacy and safety.

As for studies investigating the use of HIPEC in LAGC in a neoadjuvant setting, there is a lack of completed studies, although several ongoing trials are evaluating its efficacy and safety. The Dragon II trial is being conducted to compare the combination of neoadjuvant HIPEC plus neoadjuvant chemotherapy, subsequent surgery, intraoperative HIPEC, and adjuvant chemotherapy with surgery plus adjuvant chemotherapy alone. Neoadjuvant HIPEC will be administered in one cycle lasting 60 min at 43 degrees Celsius with 80 mg/m^2^ of paclitaxel, while both neoadjuvant and adjuvant chemotherapy will follow the SOX regimen. The study endpoints include 5-year progression-free survival, 5-year overall survival, peritoneal metastasis rate, and morbidity rate [111]. Another ongoing trial (NCT06760858) aims to investigate the efficacy and safety of HIPEC in the neoadjuvant and adjuvant settings, combined with neoadjuvant tislelizumab and the SOX regimen, in comparison with HIPEC and SOX alone. HIPEC will be administered with 120 mg of docetaxel, while tislelizumab will be provided at a dose of 200 mg. The primary endpoint is 3-year disease-free survival, with secondary endpoints including pCRR, progression-free survival, tumor regression grade, and the incidence of adverse reactions [112]. Table 3 summarizes the completed and ongoing trials on HIPEC in the setting of resectable LAGC.

### 7.2. Pressurized Intraperitoneal Aerosol Chemotherapy for Locally Advanced Gastric Cancer

PIPAC is based on fundamental physical principles: it employs the effect of a pressurized aerosol to achieve homogeneous drug distribution across the peritoneal cavity and improve tissue penetration. Pressurization enhances the solubility and diffusion of chemotherapeutic agents into cancerous tissues, thereby overcoming the limitations posed by the increased interstitial fluid pressure often observed in tumors. In addition, aerosolized drug particles provide a larger surface area for contact with peritoneal surfaces, ensuring more effective exposure of tumor cells to the chemotherapeutic agent [113]. Similar to HIPEC, PIPAC is most commonly indicated for peritoneal metastasis. A combination of 7.5 mg/m^2^ cisplatin and 1.5 mg/m^2^ doxorubicin is the most frequently used regimen, while oxaliplatin at 92 mg/m^2^ is also employed, albeit less commonly. The median number of PIPAC cycles per patient ranges from 1.5 to 3.0 [114]. Like HIPEC, systemic chemotherapy is often administered concurrently with PIPAC. PIPAC is more commonly used in the adjuvant setting, with the aim of eradicating residual microscopic disease and preventing peritoneal recurrence [115].

There is a paucity of studies specifically exploring the role of PIPAC in LAGC, which distinguishes it from HIPEC. An advanced search of publicly available databases revealed only one completed study on this topic. The PIPAC-OPC4 study evaluated the feasibility and safety of intraoperative PIPAC using 10.5 mg/m^2^ of cisplatin and 2.1 mg/m^2^ of doxorubicin in a cohort of 21 patients. While the study does not report any long-term outcomes, it documents complication rates potentially associated with PIPAC in two patients (9.5%): anastomotic leakage and duodenal blow-out. Negative peritoneal lavage cytology was the only reported measure of effectiveness and was observed in all patients [116].

According to the information available in the ClinicalTrials.gov database, four ongoing studies are evaluating the administration of PIPAC in LAGC. The DRAGON VI: PISOXO Phase I study aims to assess the efficacy and safety of neoadjuvant chemotherapy with PIPAC in combination with the SOX regimen and olaparib in a cohort of Chinese patients with locally invaded gastric cancer. The study plans to administer the first cycle of PIPAC during the initial laparoscopic exploration, followed by three cycles of SOX chemotherapy. The second cycle of PIPAC will be administered during the second laparoscopic exploration as well as gastrectomy, followed by an additional three cycles of SOX combined with olaparib. Docetaxel will be used for PIPAC administration. The primary outcome of the study is the pathological response rate, while secondary outcomes include 5-year overall survival and 3-year disease-free survival [117].

The GASPACCO study aims to evaluate the utility of adjuvant PIPAC administered to patients with high-risk LAGC to prevent the development of peritoneal carcinomatosis. High-risk LAGC is defined as T3-4, N0-3, M0, and CYT-. Patients will be randomly assigned to one of two groups: standard therapy (perioperative chemotherapy with the FLOT regimen plus D2 gastrectomy) or experimental therapy (perioperative chemotherapy with the FLOT regimen plus D2 gastrectomy combined with PIPAC, administered at 7.5 mg/m^2^ cisplatin and 1.5 mg/m^2^ doxorubicin). Treatment effectiveness will be evaluated based on 3- and 5-year overall survival, 5-year disease-free survival, and quality of life outcomes [118]. Safety will be assessed using the incidence of grade ≥ 3 adverse events and grade ≥ 3 laboratory toxicities, classified according to the National Cancer Institute Common Terminology Criteria for Adverse Events (NCI CTCAE Version 5.0) [119].

The SPECTRA study aims to investigate the safety and effectiveness of PIPAC administered to patients with locally advanced gastric cancer (Tx, Nx, CYT+, and/or peritoneal carcinomatosis index ≤ 3). According to the study protocol, all patients will receive three cycles of neoadjuvant systemic chemotherapy (administered in accordance with local protocols), interspersed with three PIPAC sessions at doses of 7.5 mg/m^2^ cisplatin and 1.5 mg/m^2^ doxorubicin. After completing the three cycles, all patients will undergo restaging followed by D2 resection. The study outcomes include disease recurrence and survival (evaluated at 5 years), tumor regression (evaluated at 2 years), patient morbidity, and health-related quality of life (evaluated at 2 years) [120]. Treatment complications will be graded according to the NCI CTCAE Version 5.0 [121].

The EPICURE study is another ongoing trial that aims to evaluate the efficacy of PIPAC combined with surgery and perioperative chemotherapy in the setting of high-risk LAGC, compared with surgery and perioperative chemotherapy alone. The study plans to enroll patients with clinical T2-4a stages, N0-3, M0, and CYT+ status, which converts to CYT− following neoadjuvant chemotherapy. PIPAC will be administered in three sessions (immediately after D2 resection, six to eight weeks postoperatively, and before the start of adjuvant systemic chemotherapy) at doses of 10.5 mg/m^2^ cisplatin and 2.1 mg/m^2^ doxorubicin. Peritoneal disease-free survival will serve as the primary endpoint, while secondary endpoints will include disease-free survival, overall survival, length of hospital stay, postoperative toxicity, complications and mortality, quality of life, and the proportion of patients requiring postoperative systemic chemotherapy [121]. Table 4 presents an overview of completed and ongoing studies evaluating the efficiency and safety of PIPAC in resectable LAGC.

**Figure 1:**
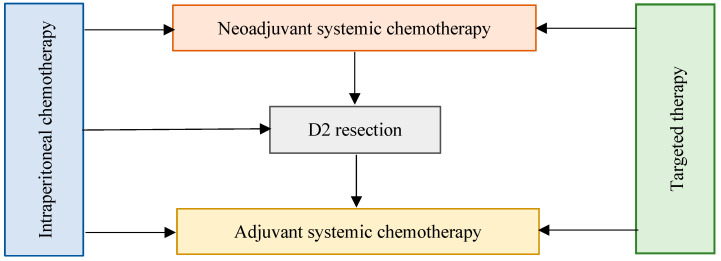
Chemotherapy options for locally advanced gastric cancer.

Figure 1 presents a flowchart illustrating multimodal treatment algorithm for LAGC, integrating targeted therapy and IPC into established treatment strategies, such as perioperative chemotherapy and D2 resection. IPC can be administered pre-, intra-, and postoperatively in conjunction with systemic chemotherapy, while targeted therapy can be administered in the pre- and postoperative settings. This schematic highlights a comprehensive, integrated approach to managing or preventing microscopic peritoneal metastases, ultimately aiming to optimize treatment outcomes.

## 8. Directions for Future Research

To reduce mortality and improve survival rates in patients with LAGC, strategies can be implemented across two primary domains: diagnostic and therapeutic. From a diagnostic perspective, the development of effective screening methods is critical for the early detection of gastric cancer. Ideally, these techniques should be minimally invasive, cost-effective, and widely accessible. The incorporation of artificial intelligence (AI) into diagnostic processes has the potential to enhance the accuracy and efficiency of screening methods by enabling the automated analysis of imaging and histopathological samples, thereby facilitating early detection and reducing diagnostic delays. Some authors argue that screening tools are most effective when based on upper endoscopy [3]. However, this assertion raises concerns, as although upper endoscopy is characterized by high accuracy, it is invasive and not cost-effective. A public health approach is essential to guide the screening process, focusing on the evaluation of appropriate screening techniques, the identification of populations at higher risk of gastric cancer, an understanding of their geographic and demographic clustering, and the determination of the most effective strategies to achieve adequate screening coverage in these groups. Such an approach ensures that more patients with gastric cancer are diagnosed at an early stage, thereby increasing the likelihood of timely and effective treatment [122].

In the therapeutic domain, there is a need to improve existing treatment modalities for LAGC. While numerous systemic chemotherapy regimens are available, the optimal timing for initiating chemotherapy and the ideal duration of treatment remain inadequately understood. Currently, the prediction and management of treatment-associated side effects are influential factors shaping therapeutic strategies. However, the clinical oncology community could benefit from advanced predictive tools that integrate epidemiological data and multi-omics research to forecast these adverse events in individual patients. Such tools would enable better personalization of chemotherapy strategies, ensuring that treatments are tailored to the specific needs of each patient. Similar to the diagnostic domain, AI has the potential to significantly enhance the precision and effectiveness of these predictive tools [5].

Another challenge in the therapeutic domain is the development of new pharmaceuticals that can serve as valuable additions or replacements to existing treatments. Targeted therapy agents represent a promising area for advancement. Current evidence suggests that ICIs, such as PD-1 inhibitors, do not fully realize their therapeutic potential for patients with gastric cancer when administered as monotherapy [3,5]. This observation has two significant implications: (1) there is a need to develop combination treatment modalities that enhance the efficacy of ICIs; and (2) novel ICIs specifically designed to effectively target gastric cancer must be developed. A variety of novel ICIs have recently been developed and are currently under investigation, including agents targeting clusters of differentiation 38 (CD38), CD39, CD73, LAG-3, T cell immunoglobulin and immunoreceptor tyrosine-based inhibitory motif domain (TIGIT), T cell immunoglobulin mucin-3 (TIM-3), and V-domain immunoglobulin suppressor of T-cell activation (VISTA) [5]. Although these emerging molecules have not yet been thoroughly investigated in the context of gastric cancer, future research should address this gap

Beyond ICIs, there is also a pressing need to identify and develop other targeted therapy agents that can inhibit pathways critical for gastric cancer progression. For instance, therapies targeting angiogenesis (e.g., VEGF inhibitors), DNA damage response mechanisms (e.g., PARP inhibitors), or other molecular pathways such as HER2 overexpression or MET amplification could provide significant therapeutic benefits [5]. Expanding research in these areas is essential to diversify the arsenal of treatment options available for patients with LAGC.

Another important issue to be addressed in the therapeutic management of LAGC is the safety and efficacy of approved anti-cancer treatments in special populations, such as pediatric patients. Although gastric cancer is rare in this population, its prognosis is less favorable than in adults [123]. This necessitates the search for optimized treatment strategies, including tailored therapeutic approaches, age-specific dosing regimens, and the evaluation of novel targeted therapies that may improve clinical outcomes. A combination of systemic chemotherapy with targeted therapy regimens represents a promising approach. For instance, integrating anti-VEGF agents with standard chemotherapy may enhance treatment efficacy and improve survival outcomes compared to chemotherapy alone in this patient population [124].

In the field of IPC for LAGC, several critical questions remain unanswered, including the optimal technique, timing, duration, and selection of chemotherapeutic agents, and overall practicality. Since HIPEC was introduced into gastric cancer treatment in the 1980s, the body of research surrounding it is more extensive compared to PIPAC. Current evidence, derived predominantly from small-scale clinical trials, suggests that HIPEC is unlikely to provide additional benefits for patients with LAGC. However, the limited sample sizes and methodological variability of these studies underline the need for larger, randomized controlled trials to reach definitive conclusions about the efficacy of HIPEC in this setting. In contrast, the evidence base for PIPAC remains sparse, with a paucity of completed studies. Consequently, well-designed, large-scale clinical trials are essential to evaluate the potential benefits of PIPAC in the management of LAGC.

## 9. Conclusions

Gastric cancer remains an unresolved oncological challenge, contributing substantially to cancer-related mortality worldwide. Early detection remains critical, but detection of the disease at the stage of LAGC offers promise for improved survival outcomes and, in select cases, even curative potential. Managing LAGC requires a multimodal approach that integrates neoadjuvant and adjuvant chemotherapy, with D2 resection playing a central role. Targeted therapy represents a promising and innovative addition to the treatment landscape for LAGC. Several pharmaceuticals are currently undergoing investigation, focusing on agents that inhibit molecular pathways implicated in tumor growth and immune evasion, such as ICIs, HER2-targeted therapies, and angiogenesis inhibitors. These therapies have the potential to enhance treatment efficacy when combined with standard chemotherapy regimens. IPC is another method of chemotherapeutic agent delivery that is currently under investigation. It can be administered in the neoadjuvant, intraoperative, and adjuvant settings. When implemented in the neoadjuvant setting, IPC increases the likelihood of achieving R0 resection by reducing tumor burden and improving surgical feasibility. Administered intraoperatively, IPC minimizes the risk of peritoneal dissemination during surgical manipulation, addressing a critical pathway for metastasis. In the adjuvant setting, IPC targets microscopic residual disease to prevent the development or recurrence of peritoneal metastases, which are a major contributor to the poor prognosis associated with LAGC.

Further high-quality, randomized controlled trials are essential to optimize current chemotherapy regimens and integrate targeted therapies and IPC into routine practice. These studies should aim to improve long-term outcomes, including overall survival, disease-free survival, and health-related quality of life, thereby tailoring treatment strategies to better meet the needs of patients with LAGC.

## Figures and Tables

**Table 1:** The main perioperative chemotherapy regimens for locally advanced gastric cancer.

Trial [Reference]	Regimen	Composition and Dosage	Number of Cycles
Triplet regimens			
MAGIC [46]	ECF	50 mg/m^2^ epirubicin and 60 mg/m^2^ cisplatin, and 200 mg/m^2^ fluorouracil	3 preoperative and 3 postoperative
FLOT4-AIO [47]	FLOT	50 mg/m^2^ docetaxel, 85 mg/m^2^ oxaliplatin, 200 mg/m^2^ leucovorin, and 2600 mg/m^2^ 5-fluorouracil	4 preoperative and 4 postoperative
Tian et al. [55]	DOX	60 mg/m^2^ docetaxel, 130 mg/m^2^ oxaliplatin, and 1000 mg/m^2^ capecitabine (administered in two doses by 500 mg/m^2^)	4 preoperative
Li et al. [56]	FOFLOX	130 mg/m^2^ oxaliplatin, 400 mg/m^2^ leucovorin, and 5-ftoruracil at 400 mg/m^2^ as a bolus followed by continuous infusion at 2500 mg/m^2^	2–4 perioperative
MATCH [61]	DOS	60 mg/m^2^ docetaxel, 100 mg/m^2^ oxaliplatin, and 40–60 mg/m^2^ S-1 (depending on body surface area)	4 preoperative and 4 postoperative
Ferri et al. [65]	DCF	75-mg/m^2^ docetaxel, 75-mg/m^2^ cisplastin, and 750-mg/m^2^/day 5-fluoracil	3 preoperative and 3 postoperative
Kalachand et al. [70]	EOX	50 mg/m^2^ epirubicin, 130 mg/m^2^ oxaliplatin, and 625 mg/m^2^ capecitabine twice daily	3 preoperative and 3 postoperative
Doublet regimens			
CLASSIC [52]	XELOX	130 mg/m^2^ oxaliplatin and 1000 mg/m^2^ capecitabine	8 postoperative cycles
Dragon III [64]	SOX	130 mg/m^2^ oxaliplatin and 40–60 mg/m^2^ S-1 (depending on body surface area)	3 preoperative

**Table 2:** The main combination chemotherapy and targeted therapy regimens for locally advanced gastric cancer.

Trial [Reference]	Agent, Dosage	Pharmacological Group	Chemotherapy Regimen(s)
Peng et al. [76] Hofheinz et al. [77] Wagner et al. [78]	4–8 mg/kg trastuzumab	Human epidermal growth factor receptor 2 inhibitors	XELOX FLOT FLOT, FOLFOX, XELOX
Chai et al. [80] Shen et al. [81]	2.0–2.5 mg/kg disitamab		S-1 XELOX
Lin et al. [82] Zhong at al [83] Li et al. [84]	200 mg camrelizumab	Immune checkpoint inhibitors	S-1 plus paclitaxel SOX
Peng et al. [76] Verschoor et al. [85] DANTE trial [86]	840–1200 mg atezolizumab		XELOX DOX FLOT
Shitara et al. [87] Kennedy et al. [88]	200 mg pembrolizumab		Cisplatin-based doublet regimen, FLOT Capecitabine
Yuan et al. [89] Zhao et al. [90]	240 mg toripalimab		SOX, XELOX
Cheng et al. [91] Toji et al. [92]	360 mg nivolumab		SOX Oxaliplatin-based
TACTIC trial [93] NCT06459921 [94] NCT04065282 [95]	200 mg sintilimab		SOX FLOT, SOX XELOX
Zhang et al. [51] Lin et al. [82] Li et al. [96]	500 mg/m^2^ apatinib	Vascular endothelial growth factor inhibitors	FLOT S-1 plus paclitaxel SOX
Yu et al. [97] Rayes et al. [98] Ma et al. [99]	5–7.5 mg/kg bevacizumab		Docetaxel plus cisplatin plus capecitabine Docetaxel plus oxaliplatin Docetaxel plus oxaliplatin plus 5-fluoracil
NEO-CLAUD trial [101]	600–800 mg/m^2^ zolbetuximab	Claudin18.2 inhibitors	DOS

**Table 3:** Studies reporting the use of hyperthermic intraperitoneal chemotherapy in locally advanced gastric cancer.

Study, Reference	Status	HIPEC * Setting	HIPEC Chemotherapeutic Agent (s)	Systemic Chemotherapy Regimen (s)
Yu et al. [105]	Completed	Adjuvant	Cisplatin	SOX
Fan et al. [106]				
Huang et al. [107]				Cisplatin
Reutovich et al. [108]			Cisplatin plus doxorubicin	Not specified
Beeharry et al. [109]	Ongoing	Neoadjuvant, intraoperative	Paclitaxel	SOX
NCT06760858 [110]		Neoadjuvant, adjuvant	Docetaxel	SOX plus tislelizumab

* HIPEC—hyperthermic intraperitoneal chemotherapy.

**Table 4:** Studies reporting the use of pressurized intraperitoneal aerosol chemotherapy in locally advanced gastric cancer.

Study Title, Reference	Status	PIPAC * Setting	PIPAC Chemotherapeutic Agent(s)	Systemic Chemotherapy Regimen(s)
PIPAC-OPC4 [116]	Completed	Intraoperative	Cisplatin and doxorubicin	FLOT
DRAGON VI: PISOXO [117]	Ongoing	Neoadjuvant	Docetaxel	SOX
GASPACCO [118]		Adjuvant	Cisplatin and doxorubicin	FLOT
SPECTRA [120]		Neoadjuvant		Not specified
EPICURE [121]		Adjuvant		

* PIPAC—pressurized intraperitoneal aerosol chemotherapy.

## Data Availability

This review article relied on the analysis of data from publicly accessible databases.

## Abbreviations

The following abbreviations are used in this manuscript:

ACRG	Asian Cancer Research Group
AI	Artificial Intelligence
CAPOX	Capecitabine, Oxaliplatin
CD	Cluster of Differentiation
CIN	Chromosomal Instability
CLDN18.2	Claudin-18 isoform 2
CTLA-4	Cytotoxic T-Lymphocyte-associated Antigen 4
CYT	Cytology status of peritoneal fluid
DCF	Docetaxel, Cisplatin, 5-fluorouracil
DOS	Docetaxel, Oxaliplatin, S-1
DOX	Docetaxel, Oxaliplatin, Capecitabine
EBV	Epstein–Barr virus
ECF	Epirubicin, Cisplatin, 5-fluorouracil
FGFR	Fibroblast Growth Factor Receptor
FLOT	5-fluorouracil, Leucovorin, Oxaliplatin, Docetaxel
FOLFOX	5-fluorouracil, Oxaliplatin, Leucovorin
GS	Genomically Stable
HER2	Human Epidermal Growth Factor Receptor 2
HIPEC	Hyperthermic Intraperitoneal Chemotherapy
HR	Hazard Ratio
ICIs	Immune Checkpoint Inhibitors
IPC	Intraperitoneal Chemotherapy
LAGC	Locally Advanced Gastric Cancer
LAG-3	Lymphocyte Activation Gene-3
MET	Mesenchymal–Epithelial Transition factor
MSI	Microsatellite Instability
MSS	Microsatellite-Stable
mTOR	mammalian Target Of Rapamycin
PD-1	Programmed cell Death protein 1
PIPAC	Pressurized Intraperitoneal Aerosol Chemotherapy
pCRR	pathological Complete Response Rate
R0	Resection with negative microscopic margins
SOX	S-1, Oxaliplatin
TCGA	The Cancer Genome Atlas
TP53	Tumor Protein 53
VEGF	Vascular Endothelial Growth Factor
WHO	World Health Organization
XELOX	Capecitabine, Oxaliplatin
95% CI	95% Confidence Interval
